# Prohibitin participates in the HIRA complex to promote cell metastasis in breast cancer cell lines

**DOI:** 10.1002/2211-5463.12966

**Published:** 2020-09-21

**Authors:** Xiaoqing Huang, Jinji Liu, Qinghui Ma

**Affiliations:** ^1^ Department of Oncology and Hematology The Second People's Hospital of Foshan (Affiliated Foshan Hospital of Southern Medical University) China

**Keywords:** breast cancer, EMT, HIRA, prohibitin

## Abstract

Prohibitin (PHB) is a highly conserved, ubiquitously expressed, multifunctional protein with a well‐characterized function as a chaperone‐stabilizing mitochondrial proteins. Recently it was reported that nuclear PHB participates in HIRA chaperone complexes and regulates downstream gene expression via cell cycle independent deposition of H3.3 into DNA. However, the role of PHB in cancer progression remains controversial with conflicting reports in the literature, perhaps due to its cell type‐dependent subcellular localization. Here, we report that the increased expression of nuclear PHB is positively correlated with metastasis of breast cancer cell lines. We showed PHB participates in the HIRA complex by interacting with HIRA through the linker region of the PHB domain and stabilizes all components of the HIRA complex in breast cancer. Overexpression of nuclear PHB resulted in a higher enrichment of histone H3.3 deposited by the HIRA complex at the promoters of mesenchymal markers. This coincided with an increased gene expression level of these markers, and induced EMT in breast cancer. Overall, these molecular and structural mechanisms suggest that nuclear PHB could hold promise as a potential target for cancer therapy.

## Graphical abstract

Prohibitin (PHB) is a multifunctional and well‐characterized chaperone‐stabilizing mitochondrial protein. We report a positive correlation of increased nuclear PHB expression with breast cancer cell metastasis. We demonstrate that nuclear PHB interacts with HIRA and stabilizes HIRA complex components, enabling it to regulate metastasis in breast cancer by H3.3 deposition at mesenchymal marker promoters.

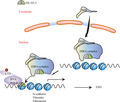

**Abbreviations**
CABIN1: calcineurin binding protein 1
CDS: coding sequence
Co‐IP: immunoprecipitation
EMT: epithelial‐to‐mesenchymal transition
hESCs: human embryonic stem cells
HIRA: histone regulator A
NES: nucleus export signal
PHB: prohibitin
PIC: protease inhibitor cocktail
PMSF: phenylmethylsulfonyl fluoride
POLII: RNA polymerase II
UBN1: ubinuclein 1

Prohibitin 1 (PHB) is a highly evolutionarily conserved protein. The amino acid sequences of mouse and rat PHB are identical and differ from human PHB in only one single amino acid. Beyond its well‐characterized function as chaperones in the mitochondrion to stabilize the mitochondrial proteins [1, 2], PHB expression level is increased in cervix, breast, and lung cancers and is a potential therapeutic target in neuroblastoma and lung cancer [3, 4, 5, 6, 7]. However, the role of PHB in cancer progression remains controversial. Sato *et al*. reported that overexpression of PHB inhibits prostate tumor growth while siRNA treatment promotes tumor growth [8]. However, Sievers *et al*. [9] showed that PHB shRNA reduces the proliferation of cultured cancer cell lines. Moreover, it has been reported that PHB is required for cancer cell migration [10]. Many conflicting results about the role of PHB have been published, and this may be explained by its different subcellular localizations. At the subcellular level, PHB localizes to the mitochondrial inner membrane, cytoplasm, nucleus, and/or plasma membrane depending on the cell type [5, 11, 12]. As subcellular localizations may influence the multiple functions within the cell, further studies are required to elucidate the different roles of the specific subsets of PHB.

Notably, structural information regarding PHB will be useful for understanding the underlying molecular mechanisms. PHB contains an N‐terminal transmembrane domain, a highly conserved PHB domain that has been suggested to be essential for protein‐protein interactions, a coiled‐coil structure of a‐helixes, and a nuclear export signal sequence consisting of a leucine‐rich NES motif at the C‐terminal end [13]. Regrettably, researchers have not yet overcome the difficulties in the crystallization of PHB and its domains. Further efforts are still required to elucidate the three‐dimensional structure of PHB.

It has been recently reported that nuclear PHB participates in HIRA complexes and regulates downstream gene expression [14]. The HIRA chaperone complex is composed of HIRA, UBN1, and CABIN1 [15, 16, 17, 18], and it is responsible for cell cycle independent deposition of H3.3 into DNA [19, 20, 21]. Histone variants H3.3A and H3.3B are expressed over the cell cycle and enriched at the active gene promoters, as well as poised regions and silent loci. Therefore, PHB involves the HIRA complex, and the HIRA complex‐facilitated H3.3 deposition may play an important role in gene regulation during the cellular process.

In this study, we focused on whether the nucleus‐localized PHB is associated with metastasis progression in breast cancer and the underlying molecular mechanism. We showed that the increased expression level of nuclear PHB was positively correlated with the metastasis of breast cancer cell lines. Additionally, we first reported that PHB interacted with HIRA through the linker region of the PHB domain and stabilized all the components of HIRA complexes in breast cancer. Forced expression of nuclear PHB increased the PHB‐involved HIRA complex‐facilitated H3.3 enrichment at the promoters of mesenchymal markers, such as N‐cadherin, vimentin, and fibronectin, and upregulated their gene expression levels through which nuclear PHB promoted the epithelial‐to‐mesenchymal transition (EMT) of breast cancer cells.

## Materials and methods

### Cell culture

The MDA‐MB‐231 and MDA‐MB‐468 cell lines (preserved in our institution) were cultured in L‐15 medium with 10% fetal bovine serum (FBS) and 80 U·mL^−1^ penicillin at 37 °C in a humidified atmosphere. The MDA‐MB‐293 cell line was cultured in DMEM with 10% fetal bovine serum (FBS) and 2 mm l‐glutamine. Cell proliferation assays were performed using Cell Proliferation Kit I—MTT (Roche, Cat. #11465007001).

### Subcellular fraction isolation

Cells cultured in 100 mm‐diameter Petri dishes were washed with PBS and the whole plate was stored at –80 °C until use. To isolate subcellular fractions, 1250 μL of lysis buffer (10 mm HEPES‐NaOH pH 7.9, 10 mm KCl, 1.5 mm MgCl_2_, and 0.5 mm β‐mercaptoethanol supplemented with protease inhibitor cocktail, PIC) were added to the dish. Next, 25 μL of 10% NP‐40 was added to the cell lysate, vortexed briefly, and centrifuged at 16 000 ***g*** for 15 min. The supernatant consisted of cytoplasmic proteins. Further, the pellet was washed with cold PBS, treated with 150 μL of nuclei lysis buffer (10 mm Tris‐HCl, pH 7.6, 420 mm NaCl, 0.5% NP‐40, 1 mm DTT, 1 mm PMSF, 2 mm MgCl_2_ plus PIC), and vortexed every 5 min for a total of 15 min. The lysate was centrifuged at 16 000 ***g*** for 15 min. The supernatant consisted of soluble nuclear proteins.

### Constructs

The full‐length CDS of *PHB* was cloned into pLVX (Novagen, Gibbstown, NJ, USA). The truncated forms of *PHB*, such as *PHB* domain (aa.55 – 172), coiled‐coil domain (aa.175 – 252), *PHB* and coiled‐coil domain (aa.55 – 252), and PHB‐D‐NES (aa.1 – 252) were also cloned. shRNA sequences for NT and *PHB* were designed, and viral was packaged as described previously. shRNA #1: gggaaggaguucacagaag. shRNA #2: cgacgaccuuacagagcga [14]. The sequences of all constructs were verified by DNA sequencing. Lentivirus was used as a gene delivery vector for transfection.

### Co‐IP and western blot analyses

Cells were lysed with the NP40 Cell Lysis Buffer (Life Technologies, Carlsbad, CA, USA) and PIC. The Co‐IP buffer consisted of 50 mm Tris, pH 7.4, 100 mm NaCl, 5 mm EDTA, 50 mm NaF, 1 mm Na_3_VO_4_, 0.1% Nonidet P40 (NP40), 0.02% NaN_3_, 1 mm PMSF, and 1× PIC. Western blot analysis was performed using conventional methods. PHB antibody was purchased from Santa Cruz Biotechnology (Dallas, TX, USA); E­cadherin and vimentin antibodies, from Cell Signaling Technology (Beverly, MA, USA), and CABIN1, UBN1, HIRA, and H3.3 from Millipore (Burlington, MA, USA). All the antibodies were used following the manufacturers’ protocols.

### Wound healing assays

Cells were cultured as a monolayer to 100% confluence and scratched with a sterile 20 μL pipette tip. Cellular migration was observed at 0 and 1 day under an inverted phase contrast microscope.

### ChIP‐qPCR assays

Cells were crosslinked with 1% formaldehyde for 15 min at 37 °C and quenched with 0.125 m glycine for 5 min at 25 °C. Cells were transferred into Eppendorf tubes containing cold PBS and 1× PIC, and centrifuged to remove the supernatant. The cell pellet was suspended in 1% SDS FA cell lysis buffer containing 50 mm HEPES‐ KOH (Sigma, St. Louis, MO, USA), pH 7.5, 150 mm NaCl, 1 mm EDTA, 1% Triton X‐100, 0.1% sodium deoxycholate, and 1% SDS supplemented with 1× PIC. Further, the pellet was rotated for 15 min at 4 °C, centrifuged at 15 000 ***g*** for 45 min, and resuspended in 1 mL of 0.1% SDS FA cell lysis buffer supplemented with 1 × PIC and 1 × PMSF, and then sonicated, followed by centrifugation at the maximum speed for 12 min. One percent of the supernatant was used as the input. Other supernatants were precleaned and incubated with the corresponding specific antibody at 4 °C overnight. Protein A and Protein G Dynabead mixture (100 μL) was used to pull down antibody‐precipitated protein‐DNA complexes for 15 min at room temperature, and then gently washed with buffer. Chromatin was dissociated from proteins and DNA was purified using the QIAquick PCR Purification Kit (QIAGEN, Hilden, Germany) as described previously [14].

### Structure analysis

The coordinates were downloaded from the Protein Data Bank and figures were prepared using PyMOL (New York, NY, USA).

## Results

### Level of nuclear PHB is higher in highly metastatic MDA‐MB‐231 breast cancer cell line compared to the poorly metastatic MDA‐MB‐468 breast cancer cell line

To investigate the relationship between nuclear PHB expression and breast cancer cell metastatic potential, we examined the expression levels of nuclear PHB in the most commonly studied triple‐negative breast cell lines, including the highly metastatic MDA‐MB‐231 breast cancer cell line and poorly metastatic MDA‐MB‐468 breast cancer cell line [22, 23]. In comparison with the MDA‐MB‐231 cell line, MDA‐MB‐468 cells exhibited more typical cobblestone‐like morphology and lower migration capability, consistent with the result showing that MDA‐MB‐468 cells have a more moderate expression of the mesenchymal markers VIMENTIN and FIBRONECTIN and higher expression of the epithelial marker E‐CADHERIN at the protein level (Fig S1A). Western blotting results revealed a similar amount of PHB protein levels in cytosolic fractions of both MDA‐MB‐468 and MDA‐MB‐231, cells while lower expression of PHB in nuclear fractions of MDA‐MB‐468 cells compared to MDA‐MB‐231 cells (Fig S1B) was observed. GAPDH was the loading control for cytosolic fractions, while H3 was that for nuclear fractions. Collectively, the results showed that the high expression level of nuclear PHB is significantly correlated with increased metastasis in breast cancers.

### Overexpression of nuclear PHB increased the migration of MDA‐MB‐468 breast cancer cell line through the epithelial‐to‐mesenchymal transition

**Fig. 1:**
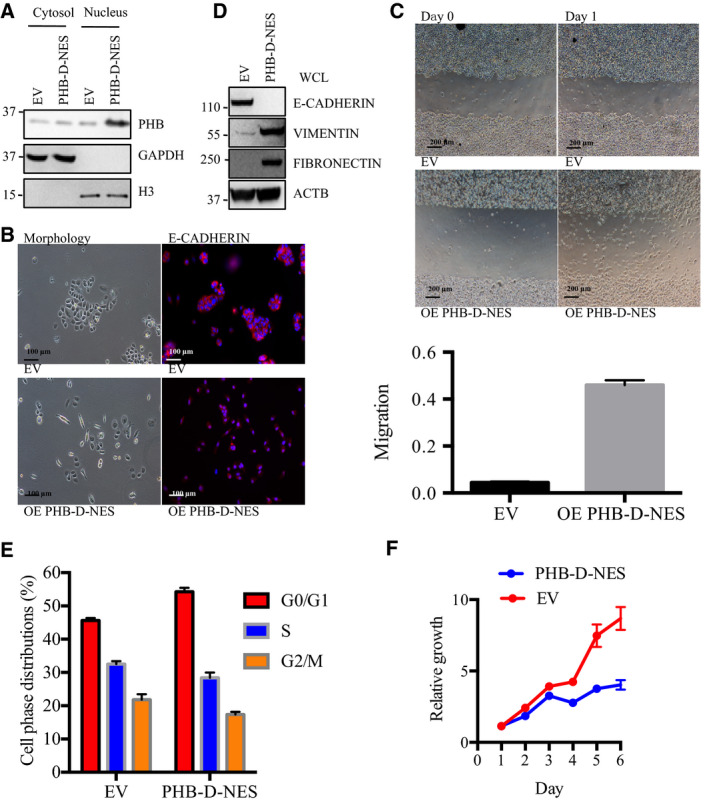
Overexpression of nuclear PHB enhances the migration of the poorly metastatic MDA‐MB‐468 breast cancer cell line through the epithelial‐to‐mesenchymal transition (EMT). (A) The cytosolic and nuclear fractions were extracted from EV‐ or PHB‐D‐NES infected MDA‐MB‐468 cells and blotted for the indicated proteins (representative of 3 biologically independent replicates). (B) Morphology of the EV‐ or PHB‐D‐NES infected MDA‐MB‐468 cells. Scale bars are 100 μm. Immunofluorescence staining of E‐CADHERIN and re‐staining with DAPI (representative of three biologically independent replicates). (C) Images of wound healing assays of EV‐ or PHB‐D‐NES infected MDA‐MB‐468 cells. Scale bars are 200 μm. The migration is ratio of area of cells migration normalized to initial total scratch region. Data are shown as the mean ± SD (standard deviation) (*n* = 3). (D) Western blots for the indicated proteins in the EV‐ or PHB‐D‐NES infected MDA‐MB‐468 cells (representative of 3 biologically independent replicates). (E) MTT cell proliferation assay of EV‐ or PHB‐D‐NES infected MDA‐MB‐468 cells. (F) Cells were stained with propidium iodide (PI) and analyzed for cell cycle phase distribution. Data are shown as the mean ± SD (*n* = 3).

To explore the role of nuclear PHB in breast cancer cell migration, we constructed a PHB‐D‐NES plasmid that expressed the PHB variant without the nuclear export signal (NES) [13]. Therefore, MDA‐MB‐468 cells infected with the PHB‐D‐NES construct only overexpressed exogenous PHB in the nucleus. Western blotting revealed a higher level of PHB proteins in the nucleus but not in the cytosolic fractions of MDA‐MB‐468 cells infected with PHB‐D‐NES than in the ones infected with empty vehicle (EV) (Fig 1A). Interestingly, MDA‐MB‐468 cells with nuclear PHB overexpression exhibited a spindle‐like morphology compared to the typical cobblestone‐like appearance of the EV‐infected MDA‐MB‐468 cells (Fig 1B). The cadherin‐mediated adherens junctions were rarely observed in the immunofluorescence images of MDA‐MB‐468 cells infected with PHB‐D‐NES construct, which demonstrated that forced expression of nuclear PHB could promote EMT transition and migration in a poorly metastatic cancer cell line (Fig 1C). In line with these cell character alterations, in MDA‐MB‐468 cells overexpressing nuclear PHB, the protein levels of the epithelial marker E‐CADHERIN significantly decreased, while those of the mesenchymal markers VIMENTIN and FIBRONECTIN were upregulated (Fig 1D). Meanwhile, the expression of nuclear PHB inhibited the cell proliferation and cell cycle progression of MDA‐MB‐468 cells (Fig 1E,F) which is consistent with previous studies[24]. Our data indicated that the overexpression of nuclear PHB could induce the EMT in breast cancer MDA‐MB‐468 cell line.

### Nuclear PHB participates in the HIRA complex in breast cancer cells

**Fig. 2:**
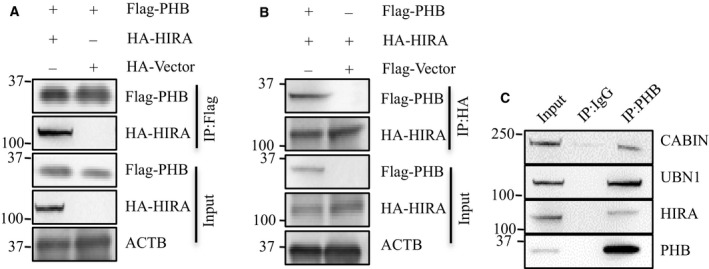
Nuclear PHB participates in the HIRA complex in breast cancer cells. (A), 293 cells were transfected with Flag‐PHB or Flag‐vector (representative of 3 biologically independent replicates). (B) HA‐HIRA or HA‐vector. Co‐IP was performed with M2 beads or anti‐HA antibody followed by western blot analysis for the indicated proteins (representative of 3 biologically independent replicates). (C) The soluble nuclear fractions were extracted from MDA‐MB‐231 cells and co‐IP was performed with anti‐PHB antibody and IgG control followed by western blot analysis for the indicated proteins (representative of 3 biologically independent replicates).

A previous study reported that nuclear PHB participates in HIRA complexes to modulate gene transcription [14]. Accordingly, we examined whether nuclear PHB was also involved in the HIRA complex and increased the transcription of the EMT‐associated genes in breast cancer cells. First, The exogenous interactions were observed in HEK 293T cells. Flag‐tagged PHB (Flag‐PHB) and HA‐tagged HIRA (HA‐HIRA) were exogenously expressed in HEK 293T cells followed by immunoprecipitation (IP) with M2 beads and α‐HA antibody, respectively. The western blot analysis results showed that Flag‐PHB precipitated HA‐HIRA, and HA‐HIRA could also pull down Flag‐PHB (Fig 2A,B). Next, we performed co‐immunoprecipitation (Co‐IP) to verify the interaction between PHB and the HIRA complex in the soluble nuclear fraction of breast cancer cells. The soluble nuclear fractions were extracted from MDA‐MB‐231 cells and immunoprecipitated with α‐PHB antibody. The endogenous HIRA, UBN1, and CABIN1 (components of HIRA complexes) were also pulled down by PHB (Fig 2C). These Co‐IP data suggested that nuclear PHB participates in the HIRA complex in breast cancer cells.

**Fig. 3:**
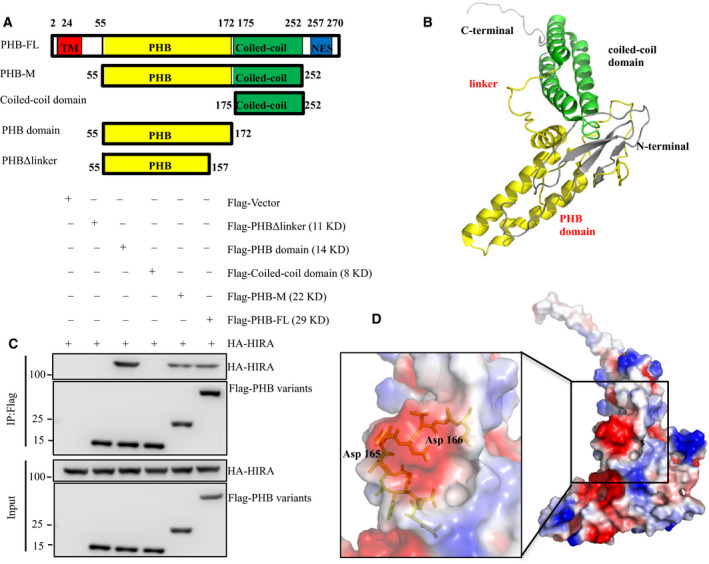
Mapping the interaction domain in PHB. (A) Schematic illustrations of PHB fragments. TM, transmembrane domain; NES, nucleus export signal. (B) Ribbon representation of the predicted structure model of PHB (PDB code: 1LU7). The PHB domain is shown in green and the coiled‐coil domain shown in yellow. The loop between PHB domain helix and coil‐coil domain is the linker region. (C) 293 cells were transfected with the indicated Flag‐tagged PHB fragments and HA‐HIRA. Co‐IP was performed with M2 beads followed by western blot analysis (representative of three biologically independent replicates). (D) A deep hydrophobic cavity in PHB is available for external interactions. The surface was colored according to electrostatic potential, with blue indicating positive and red indicating negative charge. Polar residues of the linker region stick out of the protein surface. Hydrophobic pocket are formed by the linker region.

To map the PHB domains involved in the interaction with HIRA complexes, we performed sequence and structural analyses of PHB. Previous sequence analyses showed that PHB consisted of a N‐terminal hydrophobic membrane‐anchoring or transmembrane domain, followed by the conserved PHB domain, coiled‐coil domain, and C‐terminal NES (Fig. 3A). Since no crystal structure of PHB was available in the PDB bank [25], we performed the structural analysis based on homology structure modeling. The only experimentally determined structure for the PHB superfamily was the NMR structure of mouse flotillin‐2. However, we could not use it as a template for the homology structure modeling, as the sequence similarity of PHB to mouse flotillin‐2 was only 4%. Alternatively, a theoretical model for the three‐dimensional structure of PHB using fold recognition has been deposited in the PDB bank (PDB code: 1LU7) [26]. PHB adopted an elongated scaffold shape, which may contribute to the scaffold function of the PHB protein. It mainly contained a conserved PHB domain (including several α‐helixes, β‐sheets, and a loop linker; indicated in yellow) followed by three α‐helixes coiled‐coil domain (indicated in green) in the C‐terminal (Fig. 3B).

We then defined the domain, which contributed to the interaction interface of PHB to HIRA complexes. The Flag‐PHB‐FL (full length), Flag‐PHB‐M (fragment includes PHB domain and coiled‐coil domain), Flag‐coiled‐coil, and Flag‐PHB domains were transfected into HEK 293T cells together with HA‐HIRA, followed by Co‐IP with M2 beads. Consequently, we found that Flag‐PHB‐FL, PHB‐M, and PHB domains but not the coiled‐coil domain pulled HA‐HIRA down efficiently and specifically (Fig. 3C), suggesting that the PHB domain may interact with the HIRA complex. Notably, the Co‐IP results revealed that the PHB domain‐delete‐linker (PHB ‐D‐linker) (Fig. 3A,B) no longer interacted with the HIRA complex indicating that the linker region located between the PHB and coiled‐coil domains is essential for the binding of the HIRA complex (Fig. 3C). After a detailed surface analysis of the structure model, we found that the linker region was lined with polar residues Asp165 and Asp166 sticking out of the protein surface. Moreover, the linker formed a deep hydrophobic cavity with residues in the vicinity, making it available for external interactions (Fig. 3D). Thus, nuclear PHB participated in the HIRA complex in breast cancer cells and based on the structure analysis, we concluded that the linker region of the PHB domain might be the binding site of the HIRA complex.

### PHB‐involved HIRA complex regulates EMT‐associated genes in breast cancer cell lines

**Fig. 4:**
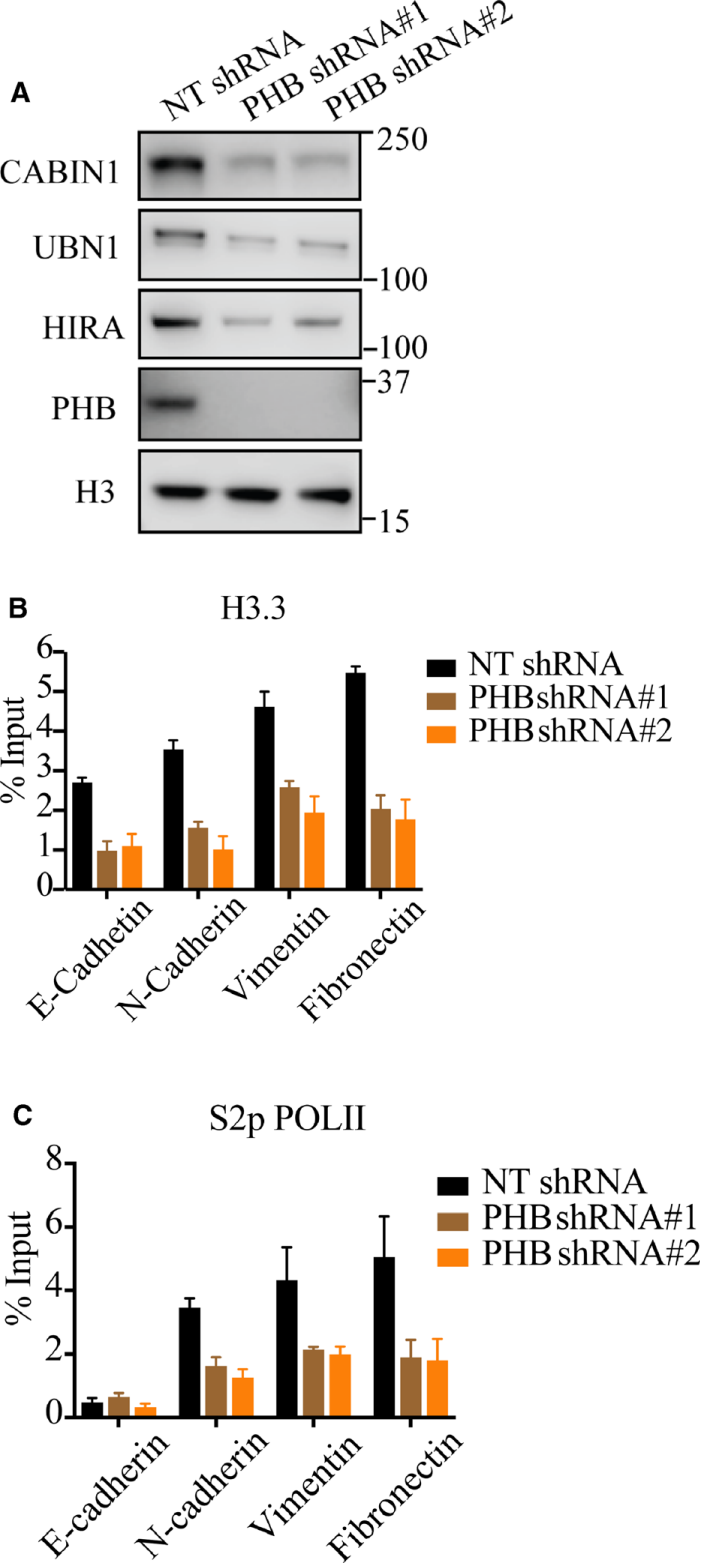
Prohibitin‐involved HIRA complex regulates EMT‐associated genes in breast cancer cell lines. (A) Knockdown of PHB in MDA‐MB‐231 cell line reduces the protein levels of HIRA, UBN1 and CABIN1. H3.3 was used as a loading control. (B,C) ChIP‐qPCR analysis for enrichment of H3.3 (B) and S2p POLII (C) at the promoters of E‐cadherin, N‐cadherin, vimentin and fibronectin genes in wild‐ type (control) and PHB shRNA‐infected MDA‐MB‐231 cell lines, respectively. Data are shown as the mean ± SD (*n* = 3) relative to the input.

A previous study of PHB in human embryonic stem cells (hESCs) demonstrated that PHB is essential for the stability of the HIRA complex and knockdown of PHB leads to decreased protein levels of all components of HIRA complexes [12], including UBN1, CABIN1, and HIRA. In the MDA‐MB‐231 cell line, we found that the knockdown of PHB using lentivirus could also reduce the protein levels of HIRA, UBN1, and CABIN1 (Fig. 4A) to that of hESCs. To further understand how PHB‐involved HIRA complexes modulate the expression levels of EMT‐associated genes in MDA‐MB‐231 cell line, we performed H3.3 and S2p POLII ChIP‐qPCR in WT (wild type) and PHB shRNA‐infected MDA‐MB‐231 cells, respectively. Our ChIP‐qPCR data showed that the deficiency of PHB in MDA‐MB‐231 cells decreased H3.3 enrichment at all these tested EMT‐associated maker genes, consistent with the HIRA complex function (Fig. 4B). Interestingly, S2p POLII ChIP‐qPCR demonstrated the decreased binding of the elongation form of RNA POLII at these mesenchymal gene promoters but not of the epithelial gene promoter in PHB deficient cells. We further confirmed that knockdown of PHB reduced the protein level of EMT markers VIMENTIN and FIBRONECTIN and inhibited the migration of the MDA‐ MB‐231 breast cancer cell line (Fig. S2) which supports the important role of PHB‐involved HIRA complex in increasing mesenchymal gene expression.

## Discussion

Although PHB has been studied extensively, the subsets of PHB from different subcellular fractions deserve further exploration as their distinct functions and pathological significances remain elusive [27]. In this study, we reported for the first time that the higher expression level of nuclear PHB is significantly associated with breast cancer metastasis. To elucidate the role of nuclear PHB, we used the PHB‐D‐NES plasmid. In a previous study, researchers identified the NES motif at the C‐end of PHB. Nuclear export of proteins is mediated by NES through nuclear pore complexes consisting of NES‐containing protein, CRM1 in conjunction, and Ran GTPase in the nucleus [28]. The export of PHB decreased by the inhibitor of the CRM1‐mediated export which led to the identification of NES within PHB. Deletion of NES blocked the export of PHB to the cytoplasm; therefore, cells infected with PHB‐D‐NES mainly overexpressed nucleus PHB and not the other subsets of PHB such as mitochondrial PHB and plasma PHB. Our further investigations showed that the overexpression of nuclear PHB would induce EMT in breast cancer cell lines and increase the metastasis ability.

Histone regulator A is a chaperone responsible for DNA‐replication‐independent H3.3 deposition on the chromatin and this has only been discovered recently [14]. Initially, HIRA‐facilitated H3.3 deposition was considered to mainly enrich at the promotors of active genes. Further studies demonstrated that HIRA‐controlled H3.3 deposition could also be associated with negative chromatin modifications as PRC2 complex and enriched in the silent regions of the genome [12]. The known components of the HIRA complex are HIRA, UBN1, CABIN1, and PHB. This is the first study to report that PHB also participates in the HIRA complex in cancer cells and PHB interacts with HIRA through the linker region of the PHB domain.

Nonetheless, additional efforts are required to obtain the crystal structure of the critical HIRA complex for a better understanding of its epigenetic chaperone function. Since knockdown of PHB would downregulate the protein levels of all components of the HIRA complex (HIRA, UBN1, and CABIN1), we proposed that PHB may play a role as a scaffold protein to stabilize the HIRA complex in the nucleus, similar to its role in the mitochondrion. Therefore, it is important to involve PHB protein in HIRA complex preparation during future crystallization.

In summary, this is the first study to elucidate the role of nuclear PHB in cancer metastasis, as well as the detailed underlying molecular and structural mechanisms. PHB participates in and stabilizes an important epigenetic chaperone HIRA complex in breast cancer cells. Accordingly, overexpression of nuclear PHB upregulates the HIRA complex‐controlled H3.3 enrichment and increases the level of mesenchymal markers and finally induces the EMT in breast cancer. Our findings will shed lights on future structure study of the HIRA complex and suggest that nuclear PHB may be a potential target for new anti‐metastasis therapy [29].

## Conflict of interest

The authors declare no conflict of interest.

## Author contributions

XH designed the study and wrote the manuscript. JL, QM, and XH performed all experiments and data analyses in the study. XH conducted structural analyses.

## Supporting information


**Fig S1.** Level of nucleus PHB is higher in highly metastatic breast cancer cell line 231 compared to the poorly metastatic breast cancer cell line 468.

**Fig S2.** Knockdown of PHB inhibits the migration of the MDA‐ MB‐231 breast cancer cell line through the inhibition of EMT.

## Data Availability

The theoretical model for the three‐dimensional structure of PHB used in this study has been published [26]. It is available in the Protein Data Bank https://www.rcsb.org/structure/removed/1LU7.
