# Supplementary material for: Prohibitin participates in the HIRA complex to promote cell metastasis in breast cancer cell lines
Source: FEBS Open Bio. 2020 Sep 21;10(10):2182–90. doi: 10.1002/2211-5463.12966 (PMC7530387; doi:10.1002/2211-5463.12966)
Supplement: Supplementary file 1 — Fig S1. Level of nucleus PHB is higher in highly metastatic breast cancer cell line 231 compared to the poorly metastatic breast cancer cell line 468. [file FEB4-10-2182-s001.pdf]

Figs 1

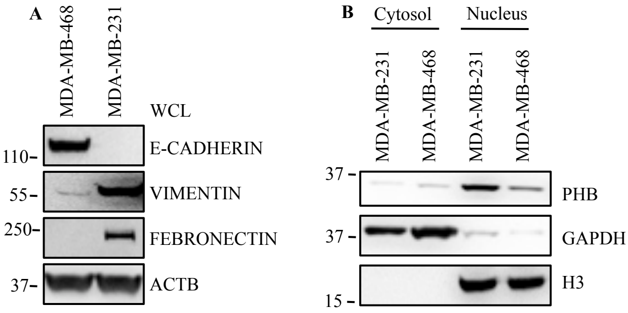

Fig s1: Level of nucleus PHB is higher in highly metastatic breast cancer cell line 231 compared to the poorly metastatic breast cancer cell line 468. A, Western blots for the indicated proteins in whole cell lysates of 468 and 231 cells. B, The cytosol and nucleus fractions were extracted from 468 and 231 cells and blotted for the indicated proteins.
