# Supplementary material for: Prohibitin participates in the HIRA complex to promote cell metastasis in breast cancer cell lines
Source: FEBS Open Bio. 2020 Sep 21;10(10):2182–90. doi: 10.1002/2211-5463.12966 (PMC7530387; doi:10.1002/2211-5463.12966)
Supplement: Supplementary file 2 — Fig S2. Knockdown of PHB inhibits the migration of the MDA‐ MB‐231 breast cancer cell line through the inhibition of EMT. [file FEB4-10-2182-s002.pdf]

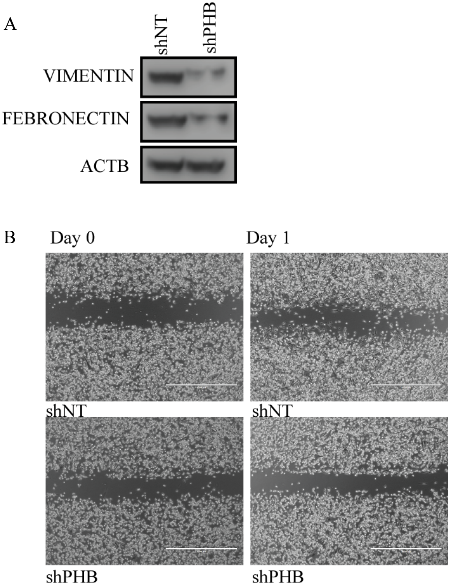

Fig s2: Knockdown of PHB inhibits the migration of the MDA- MB-231 breast cancer cell line through the inhibition of EMT. A, Western blots for the indicated proteins in the shNT or shPHB infected MDA-MB-231 cell line. B, images of wound healing assays of shNT or shPHB infected MDA-MB-231 cell line. Scale bars is 1000 µm. (representative of 3 biologically-independent replicates)
